# Drying Behavior of Bulgur and Its Effect on Phytochemical Content

**DOI:** 10.3390/foods11071062

**Published:** 2022-04-06

**Authors:** Sfayhi Terras Dorra, Dridi Farah, Hadjyahia Nesrine, Allouch Wafa, Zarroug Youkabed

**Affiliations:** Field Crop Laboratory (LR16INRAT02), National Institute of Agronomic Research of Tunisia, University of Carthage, Rue Hedi Karray, Ariana 2049, Tunisia; dridi.farah8@gmail.com (D.F.); nesrinehajyahia@gmail.com (H.N.); allouchwaf@gmail.com (A.W.); zarrougyoukabed@yahoo.fr (Z.Y.)

**Keywords:** bulgur, wholegrain, yellow berry, drying, phytochemical

## Abstract

The objective of this study was to determine the influence of two types of dryers (hot air oven and vacuum dryer) and the yellow berry percentage (1.75%, 36.25%, 43.25%) on the drying process and phytochemical content of bulgur. Results showed that the Midilli model successfully described the moisture diffusion during drying at 60 °C in all bulgur samples, where an increase in yellow berry percentage generated an increase in moisture content. Effective diffusion coefficient (D_eff_) increased significantly (*p* ≤ 0.05) from 7.05 × 10^−11^ to 7.82 × 10^−11^ (m^2^.s^−1^) and from 7.73 × 10^−11^ to 7.82 × 10^−11^ (m^2^.s^−1^) for the hot air oven and vacuum dryer, respectively. However, it decreased significantly with a decrease of yellow berry percentage. It was concluded that the vacuum dryer provided faster and more effective drying than the hot air oven. Total polyphenol (TPC), total flavonoid (TFC), and yellow pigment contents (YPC) of bulgur were investigated. TPC ranged between 0.54 and 0.64 (mg GAE/g dm); TFC varied from 0.48 to 0.61 (mg QE/g dm). The YPC was found to be between 0.066 and 0.079 (mg ß-carotene/100g dm). Yellow berry percentage positively and significantly affected the TPC, TFC, and YPC contents due to the hard separation of the outer layers from the starchy grain during the debranning step.

## 1. Introduction

Bulgur is a famous dish in Central Asia, Turkey, the Middle East, and North Africa [1]. It is considered a highly nutritious food [2], since it contains high dietary fiber content [3,4] and a high amount of vitamins and minerals such as phosphorus, zinc, potassium, and selenium. It also has a low glycemic index [5]. Bulgur is composed of 9–13% water, 10–16% protein, 1.2–1.5% fat, 76–78% carbohydrate, 1.2–1.4% ash, and 1.1–1.3% fiber [6]. Generally, bulgur is made from hard wheat (*Triticum durum*) [7], which results in its yellow color and higher protein content compared to the other wheat types [8,9]. However, other grains can be used to produce bulgur, such as bitter and sweet lupin [10], barley [11], soybean [12], and chickpea [13]. The quality of durum wheat affects the bulgur quality. In fact, a positive correlation has been determined between bulgur yield and the thousand kernel weight [14], but no studies have shown a relationship between yellow berry percentage and bulgur quality. As known, yellow berry (yb) is a physiological disorder, mainly found in durum wheat. It is defined as the poor development of endosperm [15], where soil with nitrogen insufficiency is the main cause [16]. This disorder is characterized by starchy spots that can cover small areas up to the entire grain [17,18]. The grain becomes less vitreous, starchy, softer, opaque, and light-colored [17,19,20]. Lopez-Ahumada et al. [18] reported that grains with yellow berry have higher starch content than normal grains, which affects crystallinity percent. Dexter et al. [20] reported that yellow berry grains have high moisture and low protein content compared to normal vitreous grains. A negative correlation has been found between protein content and yellow berry percentage [21,22].

Bulgur production involves several steps: cooking, drying, tempering, and debranning [23,24,25]. Due to the importance of drying, several researchers have tried to model moisture diffusion in parboiled wheat [26,27,28] and wheat [29,30]. After cleaning, the grains are cooked in boiling water until the starch is completely gelatinized. Bayram [31] proposed 40 min as the optimum cooking time, where the starch is gelatinized without any deformation of the wheat kernel. Additionally, Sfayhi-Terras et al. [23] determined that 43 min is the ideal cooking time to generate high-quality bulgur. During cooking, time and temperature are considered the most critical parameters that have an impact on the dimensions, volume, and crease of the wheat kernel [24,32]. The boiled wheat is then dried to decrease moisture content from 45% to 10% (d.b). After drying, the dried, parboiled wheat is debranned, which involves removing the grain outer layers by abrasion and friction [33].

Since the cooking and drying operations may significantly alter the color, yield, chemical composition, nutritive quality, and physical properties of bulgur [11,13,34,35,36], many works have studied the effect of each processing step on bulgur quality. Hayta [37] investigated the effects of different drying methods (solar, sun, microwave, tray drying) on yield and protein extractability. Among the drying methods, the yield of the sun-dried sample was the lowest. However, these methods did not affect the extraction of protein. Kadakal et al. [38] studied the effect of cooking (in a beaker at 90 and 100 °C, and autoclave at 121 °C) and drying (in a hot air oven at 60, 70, and 80 °C, and in open-air sun-drying) on the water-soluble vitamins of bulgur. It was shown that hot air oven drying at 60 °C does not affect the water-soluble vitamin contents, unlike drying in sun-drying and hot air oven drying at 80 °C.

It is well-known that during the drying process, temperature directly affects the nutritional quality of products. Yilmaz and Koca [39] reported that autoclave cooking and hot air drying at 60 °C presented the highest retention of total phenolic content and total yellow pigment than both autoclave cooking/hot air drying and microwave cooking and drying. Although extensive work has been carried out on drying, limited literature is available on the variation of bioactive phytochemicals in bulgur such as total phenolic, flavonoid, and yellow pigment contents during drying, and no work was found that studied the effect of yellow berry percent on the drying behavior and quality of bulgur.

The objective of the present work is to investigate the effect of yellow berry percent and dryer type on drying behavior, to find a suitable drying model, to determine the effective diffusivity coefficient, and to study the change of the bioactive components of bulgur during the drying operation.

## 2. Materials and Methods

### 2.1. Material

For this study, bulgur was prepared from Tunisian durum wheat (*Triticum durum*), Maali variety, for one cultivation with high quality. Three different samples from this variety were used. The difference was in the yellow berry percentage (yb) and the thousand-kernel weight (TKW). The yellow berry percentages were 1.75%, 36.25%, and 43.25%, and the TKW were 53.8 g, 53.9 g, 48.6 g, respectively. The moisture, protein, and ash content of these samples were 11.0 ± 0.5% (d.b), 13.0 ± 0.4% (d.b), and 1.7 ± 0.4% (d.b), respectively. The thousand-kernel weight (TKW) was determined using the Numigral Chopin (Chopin, Villeneuve-la-Garenne, France). Yellow berry percentage was determined by inspecting 50 kernels sliced using a Pohl farinothome (Chopin, Asnières-sur-Seine, France). Moisture content before debranning was determined according to the AACC-approved method 44-15A [40]. Grain protein was evaluated using a Near-Infrared Spectroscopy System (Perten-Inframatic-8600, Hamburg, Germany) [41]. Ash content was evaluated according to ICC Standard 104/1 [42].

### 2.2. Bulgur Processing

The grain was cleaned with distilled water for 1 min to remove any adhesive particles stuck to the surface of the kernels. Then, it was cooked in boiling water at 100 °C for 42–53 min until the entire grain starch was gelatinized. The cooking time was determined using the center cutting method [31]. Precooked grain (100 g) was dried at 60 °C for 180 min. During the drying operation, 5 g was collected at 15 min intervals. Two types of natural convective air dryers were used for dehydration of precooked grain: a hot air oven (Venticell 404-ECO line, München, Germany) and a vacuum dryer (Monferrina EC50, Castell’Alfero, Italy), where the Hr of the air was fixed at 80%. After cooking, each sample was debranned with an abrasive laboratory mill (Strong-Scott, England) at a constant speed of 830 rpm for 1.6min [23,43]. The debranned grains were separated from the debranned part with a sieve of 1.04 mm set inside the apparatus. For this study, bulgur was considered the recovered sample.

### 2.3. Moisture Content

The moisture loss from the parboiled wheat during drying was determined every 15 min for 180 min. The experiments were conducted in duplicate and average values were taken. The moisture content of samples was calculated using Equation (1):

(1) $$M_{t}\;=\;\frac{(W_{0}\;+\;W_{t})\;-\;W_{f}}{W_{t}}\;*\;100$$

where W_0_ is the initial weight (g), W_t_ is the weight of the sample (g) at any drying time (t), and W_f_ is the final weight (g). M_t_ is the moisture content of the wheat samples at the different drying times.

### 2.4. Phytochemical Analysis

Before extraction, samples were ground by a grinder (CT 293 Cyclotec, Foss, Hamburg, Germany), then separated using a sieve of 0.8 mm. According to the procedure by Mau et al. [44], the phenolic compounds were extracted with 25 mL of 80% methanol using a 2.5 g sample. The extraction solvent and the sample were mixed in an orbital shaker for 30 min at ambient temperature and then stored in the dark for 24 h at 4 °C. The mixture was filtered through Ashless Wattman paper (No. 4). The filtrate obtained was concentrated under vacuum by rota-vapor (60 °C). Thus, the extracts obtained were collected, weighed, stored at 4 °C, and protected from light. For further analysis, 1 mg of the extract was dissolved in 1 mL of methanol.

#### 2.4.1. Total Polyphenol Content (TPC)

Total polyphenol content was determined according to the procedure from Dewanto et al. [45], using a modification of the Folin–Ciocalteu method. The absorbance was measured at 760 nm using a spectrophotometer (Onda V-10 Plus, Capri, Italy), and the results were expressed as milligram gallic acid equivalents per gram of sample dry matter (mg GAE/g dm).

#### 2.4.2. Total Flavonoid Content (TFC)

Total flavonoid content was determined by using the modified method from Dewanto et al. [45]. The absorbance was measured at 510 nm using a spectrophotometer (Onda V-10 Plus, Capri, Italy). The results were expressed as milligram quercetin equivalents per g of sample dry matter (mg QE/g dm).

#### 2.4.3. Yellow Pigment Content (YPC)

Yellow pigment content was determined according to the norm ISO 11052 [46]. Ten grams of samples were extracted with 50 mL water-saturated butanol (ratio 6:2). The mixture was homogenized and kept for 16 h at room temperature. Then, it was filtered in conical bottles. The absorbance was measured at 440 nm using a spectrophotometer (Onda V-10 Plus, Capri, Italy). The results were expressed as milligram beta carotene equivalents per g of sample dry matter (mg ß-carotene/100 g dm).

### 2.5. Modeling of the Drying Process

Among the mathematical models, Lewis, Henderson and Pabis, Logarithmic and Midilli models were employed to describe the drying kinetics of the parboiled wheat (Table 1).

By noting the moisture content every 15 min in the different dryers, moisture ratios and drying rates of samples were calculated by Equations (2) and (3), respectively. The drying experiments were carried out for 180 min. The simplified equation of Rayaguru and Routray [51] was used to determine the moisture ratio (MR):

(2) $$\mathrm{MR}\;=\;\frac{M_{t}}{M_{0}}$$

where M_t_ is the moisture content at any time (%) and M_o_ is the initial moisture content (%) of the samples.

The drying rate (DR) of parboiled wheat samples was calculated using Equation (3) [52]:

(3) $$\mathrm{DR}\;=\;\frac{M_{\;t+\mathrm{dt}}\;-\;M_{t}}{\mathrm{dt}}$$

where MR_t+dt_ and MR_t_ are moisture ratios at the time (t + dt) and t (dimensionless), t is the drying time (min).

### 2.6. Effective Diffusion Coefficient

The simplified solution of Fick’s diffusion was used [53]:

(4) $$\mathrm{MR}\;=\;\frac{6}{\pi^{2}}\exp(-\;\frac{D_{\mathrm{eff}}{\;*\;\pi }^{2}\;*\;t}{R_{e}^{2}})$$

where n is the positive integer, D_eff_ is the effective moisture diffusion coefficient (m^2^s^−1^), t is drying time (s), and R_e_ is the average radius of wheat (2.21 × 10^−1^ m). Equation (4) can be written in logarithmic form:

(5) $$\ln\left(\mathrm{MR}\right)\;=\;\;\ln\frac{6}{\pi^{2}}\;-\;\frac{D_{\mathrm{eff}}{\;*\;\pi }^{2}\;*\;t}{R_{e}^{2}}$$

The effective diffusion coefficient is calculated from the slope of Equation (5), which is obtained from the graph describing the change in ln (MR) values with drying time.

(6) $$\mathrm{Slope}\;=\;\frac{D_{\mathrm{eff}}{\;*\;\pi }^{2}}{R_{e}^{2}}$$

### 2.7. Statistical Analysis

Sigma plot 14.5 (Systat Software, Inc., San Jose, CA, USA) was used to present all drying data. The variance analysis (ANOVA) was executed using the significance level of (*p* < 0.05) using SPSS software (version 23.0) (IBM Software, New York, NY, USA). The results were followed with letters in case of the existence of a significant difference.

## 3. Results

### 3.1. Drying Kinetics and Modeling

**Figure 1:**
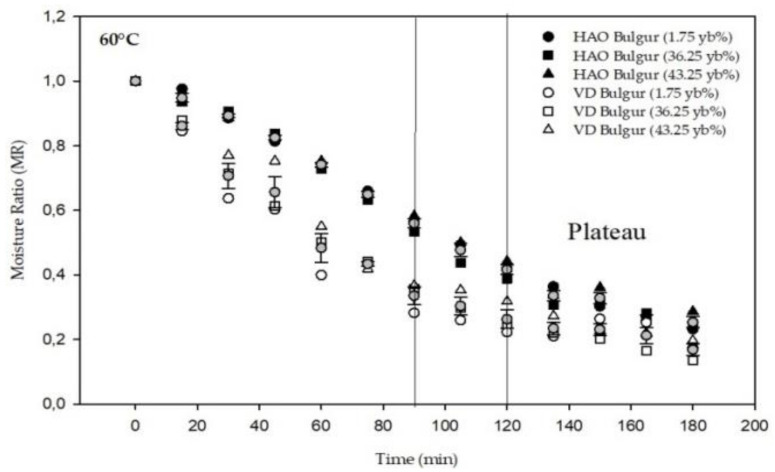
Variation of moisture ratio (MR) with time in different dryers at 60 °C. HAO: hot air oven; VD: vacuum dryer.

By noting the weight loss during the drying process, moisture ratio (MR) change over time in the different dryers was determined using Equation (2) and then presented in Figure 1. Examining Figure 1, the moisture ratio (MR) decreased with time, in both dryers, and then reached a plateau. A significant difference (*p* ≤ 0.05) was found between the two dryers, where MR was significantly lower for the vacuum dryer compared to the hot air oven. According to ANOVA results, the yellow berry percentage had a significant (*p* ≤ 0.05) effect in terms of variation of moisture ratio. The highest moisture ratio was obtained for bulgur at 43.25 yb%.

A comparison between the slopes of the drying curves for the declining phase (Figure 1) in both dryers (P_1_ hot air oven, P_2_ vacuum dryer) was realized. It can be seen from Table 2 that a significant difference (*p* ≤ 0.05) in slope values was found between oven-dried and vacuum-dried bulgur, where P_2_ was found to be the smallest. Moreover, to reach the plateau (Figure 1), the vacuum dryer required a shorter time (90 min) than the oven dryer (120 min).

However, the three vacuum-dried slopes, as well as the hot air oven-dried slopes, were found to be significantly (*p* ≤ 0.05) different (Table 3). This result indicates that the variation of yellow berry percent has a significant effect on the drying behavior of bulgur.

**Figure 2:**
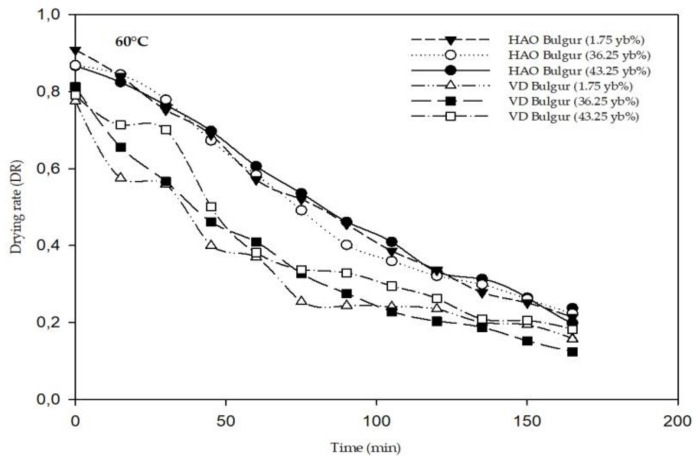
Drying rate curves in the different dryers at 60 °C. HAO: hot air oven; VD: vacuum dryer.

Using Equation (3), the drying rate (DR) variation with time was determined and is represented in Figure 2. As can be seen from Figure 2, the drying rate (DR) in both dryers decreases over time. Only one phase was noted—the falling rate period. The drying rate of vacuum-dried samples was slightly lower than the drying rate of oven-dried bulgur (Figure 2).

**Figure 3:**
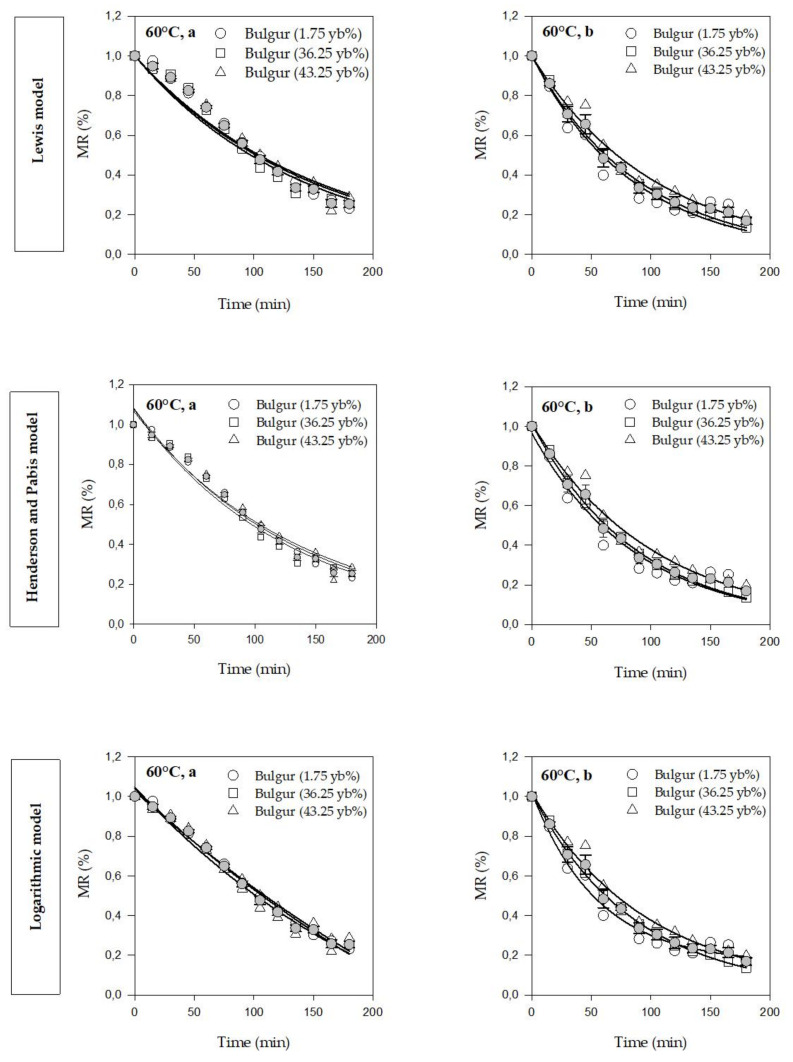

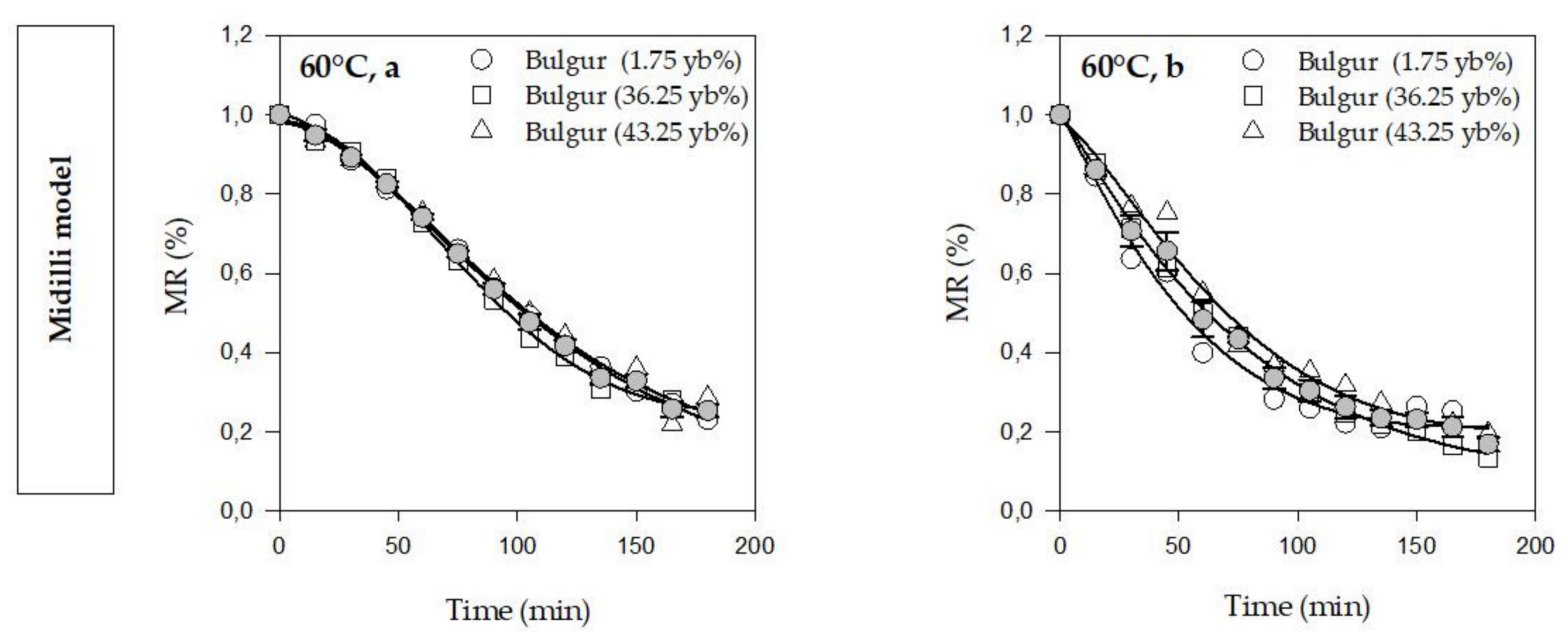
Simulated moisture ratio during drying of parboiled wheat for different dryers ((**a**): hot air oven; (**b**): vacuum dryer) at 60 °C.

The moisture ratio (MR) was fitted to the four models listed in Table 1 and presented in Figure 3. As standard error (StdErr) and residual sum of squares (RSS) values approach zero the closer the prediction is to the experimental data. The drying models were compared based on their R^2^ to assess their respective goodness of fit. Accordingly, all the tested models had high coefficient of determination (R^2^) values in the range 0.95–0.99 and 0.94–0.99 for the hot air oven and vacuum dryer, respectively. Among the used models, the Midilli model had the highest R^2^ values and the lowest StdErr and RSS values for the hot air oven and vacuum dryer as shown in Table 4.

The effect of dryer type on the drying rate constant k of the Midilli model value can be seen in Table 4. When comparing the k values of the hot air oven with the vacuum dryer, the k values increased from 6.93 × 10^−4^ to 9.51 × 10^−3^, from 4.48 × 10^−4^ to 8.24 × 10^−3^, and from 2.55 × 10^−4^ to 3.07 × 10^−3^ for bulgur 1.75 yb%, 36.25 yb%, and 43.25 yb%, respectively. Using Equation (6), the effective diffusion coefficient was determined.

Drying at 60 °C, the effective diffusion coefficient D_eff_ values varied from 6.86 × 10^−11^ ± 4.52 × 10^−21^ to 7.05 × 10^−11^ 3.17 × 10^−22^ (m^2^.s^−1^), and from 7.73 × 10^−11^ ± 4.74 × 10^−22^ to 7.82 × 10^−11^ ± 7.05 × 10^−22^ (m^2^.s^−1^) for the hot air oven and the vacuum dryer, respectively (Table 5). A significant difference (*p* ≤ 0.05) in the values of the effective diffusion coefficient values was found where the vacuum dryer presented the highest D_eff_ values (Table 5).

Meanwhile, drying at the temperature of 60 °C, the ANOVA showed that yellow berry percentage also significantly affects the effective diffusion coefficient (Table 6). An increase in yellow berry percentage generates a decrease in D_eff_ value.

### 3.2. Phytochemicals Content of Bulgur

**Figure 4:**
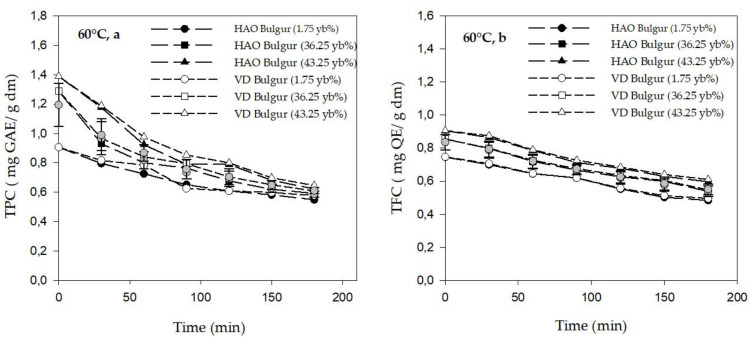
Total polyphenol content (**a**) total flavonoid content (**b**) in bulgur samples at 60 °C. HAO: hot air oven; VD: vacuum dryer.

The variation of total polyphenol content (TPC) and total flavonoid content (TFC) in bulgur during drying is presented in Figure 4a,b, respectively. It can be seen that TPC and TFC decreased over time during drying at 60 °C. After 3 h of drying at 60 °C, the TPC varied from 0.57 ± 3.20 × 10^−5^ to 0.62 ± 5.5 × 10^−4^ (mg GAE/g dm), and from 0.54 ± 3.46 × 10^−4^ to 0.64 ± 1.9 × 10^−5^ (mg GAE/g dm) for the hot air oven and vacuum dryer, respectively. The TFC of bulgur ranged from 0.48 ± 4.5 × 10^−4^ to 0.59 ± 9 × 10^−5^ (mg QE/g dm) and from 0.49 ± 6.9 × 10^−5^ to 0.61 ± 1.11 × 10^−4^ (mg QE/g dm) for the hot air oven and vacuum dryer, respectively. During drying, no significant difference was determined between the two drying methods.

**Figure 5:**
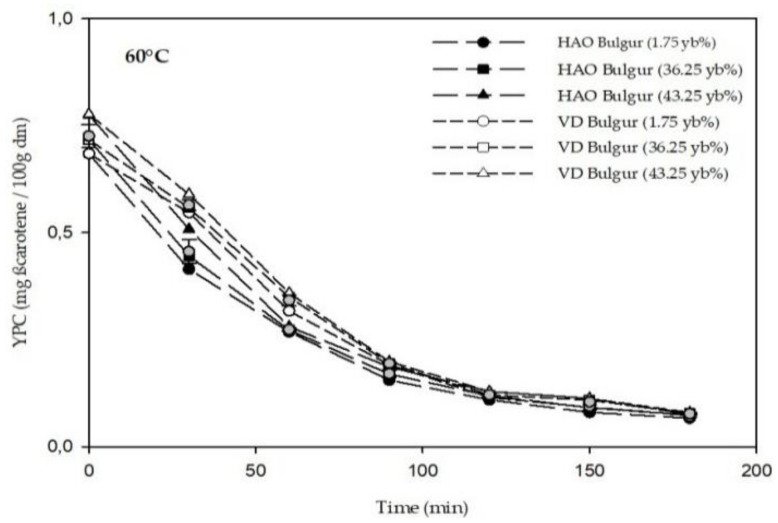
Total yellow pigment content in bulgur samples at 60 °C. HAO: hot air oven; VD: vacuum dryer.

Examining Figure 5, the yellow pigment content decreased over time. Comparing YPC in the different dryers, no significant difference was found. The YPC ranged from 0.066 ± 0.419 to 0.075 ± 1.5 × 10^−4^ (mg ß-carotene/100g dm), and from 0.073 ± 1.9 × 10^−5^ to 0.079 ± 3.09 × 10^−4^ (mg ß-carotene/100g dm) for the hot air oven and vacuum dryer, respectively.

According to ANOVA results, yellow berry percentage had a positive significant effect on the TPC, TFC, and YPC values (*p* ≤ 0.05) where bulgur 43.25 yb% samples, in the vacuum dryer and hot air oven, had the highest TPC, TFC, and YPC contents, whereas the bulgur 1.75 yb% samples presented the lowest contents.

## 4. Discussion

Drying is an important step in bulgur processing since it directly affects the quality [37]. Traditionally, bulgur is spread onto a flat surface and left to dry under the sun for 8–10 h to decrease moisture content from 45 to 10% (dry basis). Several drying methods have been studied, such as the microwave drying method [37]. However, when this technique is assisted by spouted bed drying, the bulgur has a more porous microstructure and lower water absorption capacity, inducing a decrease in drying time [54]. Savas and Basman [35] used infrared treatment at various power levels and periods as an alternative bulgur-drying technique. The results showed that infrared dried samples were similar to sun-dried samples in terms of quality, but that drying time is shorter, thus indicating that infrared drying is a promising technique for the future.

The present paper examines the drying behavior of bulgur using hot air and vacuum dryers. The drying curves obtained from the variation of moisture ratios with time were found to be similar to the drying curves observed by Yildirim [28], who established general equations describing the moisture ratio of parboiled wheat during drying at different temperatures for the different dryers used. Concerning the variation of drying rate with time, only one phase was noted: the falling rate period. The absence of a constant drying period was also reported for parboiled wheat drying in Mohapatra and Rao [27] and Yildirim [28]. Thus, the entire drying process only takes place during the falling rate period, which indicates that moisture diffusion was the governing factor [55] for deciding the drying behavior of bulgur. Comparing the two dryers, the results have confirmed that the vacuum dryer provides faster drying compared to the hot air oven dryer where the Midilli model successfully predicted the drying behavior of bulgur. The vacuum dryer showed the highest D_eff_ value, which can be explained by the easy evaporation of moisture and a higher mass transfer, confirming a faster drying behavior. This result is in agreement with Yildirim [28], who showed that the vacuum dryer was found to be the fastest compared to the convective air and forced-air dryers, and drying time was shortened with the increase of temperature. This confirms that vacuum dryers tend to work faster than other drying methods, reducing the processing time [56].

On the other side, the results have shown that yellow berry disorder had a significant effect on the drying behavior of bulgur, where a higher percent induces high MR content, low value from the Midilli model drying rate constant k, and low value of D_eff_. This result could be explained by the fact that yellow berry disorder induces a high starch content in wheat where the starchy granules are reported to have a larger diameter and high crystallinity percent than vitreous grains [18,57]. A positive correlation was found between the gelatinization enthalpy (ΔH) and crystallinity percent [58]. The ΔH exhibits the loss of the molecular double helical [59], which induces the stability of the structure and enhances the resistance of the granules to gelatinization [60]. Thus, excess water absorption is required to destabilize the structure generating the gelatinization of the starches [61]. Hence, high yellow berry percentage generates higher moisture content, which induces slower drying. As a result, the drying rate constant and the effective diffusion coefficient decrease with the increase of yellow berry percentage.

Bulgur is considered a practical food [2] since it contains several bioactive compounds. Many works have reported the presence of ferulic acid, gallic acid, 3.4hydroxybenzoic acid, epicatechin, caffeic acid, p-hydroxybenzoic acid, p-coumaric acid, syringic acid, and low amounts of chlorogenic acid in bulgur [2,39,62,63]. The effect of drying on phytochemical content is studied in this work. The obtained values of our samples are in agreement with studies by Caba et al. [2] and Ertas [64]. In fact, Caba et al. [2] investigated the composition of bioactive components of industrial bulgur samples in which the TPC varied between 0.553 and 0.621 (mg GAE/g dm), whereas Ertas [64] studied twelve industrial bulgur samples, four homemade bulgur samples produced in Turkey, and one laboratory-made sample. TPCs of the industrial, homemade, and laboratory-made bulgur samples were found to be between 0.449 and 0.968, 0.632 and 1.173, and 0.986 (mg GAE/g dm), respectively. Concerning the flavonoid contents, the obtained values were lower than those reported in Yüksel et al. [65] where the flavonoid content of bulgur flour was found to be 105.88 (mg catechin/100 g sample). This decrease in TFC in the bulgur samples might be due to the difference in wheat species used, the different bulgur production techniques, and the use of quercetin instead of catechin. In fact, according to Morel et al. [66], the catechin had a bigger effect than quercetin.

It is important to note that the values of TPC and TFC of bulgur are lower compared to wholegrain wheat since these compounds are mainly localized in the bran of durum wheat, and bulgur is defined as a debranned precooked wheat grain [67,68,69,70,71,72].

Carotenoid content in wheat bran was higher than endosperm [68], since the yellow pigments are more concentrated in the outer layers than the inner layers [73]. Lutein is the major and predominant carotenoid and is responsible for the bulgur’s distinct yellow color [74,75]. Other carotenoids, such as zeaxanthin, b-cryptoxanthin, and ß-carotene were also found [76]. A significant correlation was found between yellow pigment content (YPC) in bulgur and cultivar cooking methods as well as their interactions [36]. The moisture content of wheat and abrasion time was also found to significantly affect the total carotenoid content [77]. The carotenoid pigment and lipoxygenase activity are responsible for b* of the grain [23]. Therefore, due to the Maillard reaction, in the presence of heat applied at cooking and drying treatments, the pigments are degraded, which generates discoloration of the bulgur [78]. The obtained results are slightly higher than what was reported in the study of A.K. Elvice and Hazim Ozkaya [36], where the average YPC in coarse and fine bulgur samples was 3.14 (µg/g). This difference is probably due to the different wheat species and different bulgur production processes.

Thermal treatments, such as drying, have been reported to negatively affect the phytochemical content (polyphenol, flavonoid, and carotenoid) in bulgur, which causes its decrease [39,79]. It is important to mention that despite this decrease, no significant difference was found between the two drying methods. This might be due to the use of the same low temperature of 60 °C, which was reported to have the highest retention of total phenolic and yellow pigment content in the bulgur [25,39].

The effect of yellow berry percent on the phytochemical content can be explained by the difference in the hardness structure of the endosperm. In fact, the debranning process removes only the outer layers of the grains, which allows the recovery of intact kernels. Due to the high starch content (high yellow berry percentage), the texture becomes soft [17,19] and therefore could affect the peeling of the outer layers of grain where they are not totally removed from the grain, compared to those debranned from grains with low yellow berry percentage [17]. Therefore, the presence of the outer layers induces high TPC, TFC, and YPC contents in bulgur during debranning.

## 5. Conclusions

In this study, two different drying methods (hot air oven and vacuum drying) were used in bulgur production using three durum wheat samples at different yellow berry percentages (1.75%, 36.25%, and 43.25%). The drying behavior of the bulgur was successfully described by the Midilli model. Comparing both dryers, a significant difference (*p* ≤ 0.05) was found in terms of the variation of moisture ratio and drying rate over time. The vacuum dryer presented the highest D_eff_ and k values, confirming faster and more effective drying than the hot air oven. Yellow berry percentage had a significant effect (*p* ≤ 0.05) on the bulgur’s drying behavior. Results showed the presence of a strong correlation between high starch content and moisture content, where an increase in yellow berry percentage generates an increase in MR and a decrease in D_eff_ and k values.

Drying at 60 °C decreases bulgur phytochemical content, where no significant difference was observed between the two types of dryers. However, yellow berry disorder had a positive effect on preserving the phytochemical content in bulgur because theyremainin²the outer layers after the debranning process and induce a higher bulgur quality.

## Figures and Tables

**Table 1:** Mathematical models used.

Model Name	Model Equation	Reference
Lewis	$\mathrm{MR}\;=\;\exp\left(-\mathrm{kt}\right)$	[47]
Henderson and Pabis	$\mathrm{MR}\;=\;a\;*\;\exp\left(-\mathrm{kt}\right)$	[48]
Logarithmic	$\mathrm{MR}\;=\;a\;*\;\exp\left(-\mathrm{kt}\right)\;+\;b$	[49]
Midilli	$\mathrm{MR}\;=\;a\;*\;\exp\left(–{\mathrm{kt}}^{n}\right)\;+\;\;\mathrm{bt}$	[50]

**Table 2:** The effect of dryer type on the drying curves at 60 °C.

	Slope	Bulgur 1.75 yb%	Bulgur 36.25 yb%	Bulgur 43.25 yb%
Hot air oven	P_1_	−5.51 × 10^−3^ ± 1.98 × 10^−5 a^	−5. 35 × 10^−3^ ± 0.68 × 10^−5 a^	−4.71 × 10^−3^ ± 5.94 × 10^−5 a^
Vacuum dryer	P_2_	−7.48 × 10^−3^ ± 2.85×10^−5 b^	−6.97 × 10^−3^ ± 1.46 × 10^−5 b^	−6.56 × 10^−3^ ± 7.90 × 10^−5 b^

Mean values with a row followed by different letters are significantly different (*p* < 0.05).

**Table 3:** The effect of yellow berry percent on the drying curves at 60 °C.

	Hot Air Oven (P_1_)	Vacuum Dryer (P_2_)
Bulgur 1.75 yb%	−5.51 × 10^−3^ ± 1.98 × 10^−5 c^	−7.48 × 10^−3^ ± 2.85 × 10^−5 c^
Bulgur 36.25 yb%	−5.35 × 10^−3^ ± 0.68 × 10^−5 b^	−6.97 × 10^−3^ ± 1.46 × 10^−5 b^
Bulgur 43.25 yb%	−4.71 × 10^−3^ ± 5.94 × 10^−5 a^	−6.56 × 10^−3^ ± 7.90 × 10^−5 a^

Mean values with a row followed by different letters are significantly different (*p* < 0.05).

**Table 4:** Parameters of the four drying models.

Samples	Model	Hot Air OvenDrying				Vacuum Drying			
		Parameters	R^2^	Std Err	RSS	Parameters	R^2^	Std Err	RSS
Bulgur 1.75 yb%	Lewis	K 6.82 × 10^−3^	0.9570	0.0004	0.0381	K 1.17 × 10^−2^	0.9486	0.0599	0.0431
	Henderson and Pabis	K 7.64 × 10^−3^ a 1.08	0. 9760	0.0440	0.0213	K 1.13 × 10^−2^ a 9.95 × 10^−1^	0.9512	0.0610	0.0409
	Logarithmic	K 2.23 × 10^−3^ a 2.43 b −1.41 × 10^−1^	0.9937	0. 0236	0. 0056	K 1.80 × 10^−2^ a 8.60 × 10^−1^ b 1.59 × 10^−1^	0.9742	0.0465	0.0216
	Midilli	K 6.93 × 10^−4^ a 1.00 b 3.30 × 10^−4^ n 1.51	0.9943	0.0324	0.0095	K 9.51 × 10^−3^ a 1.00 b 9.72 × 10^−4^ n 1.12	0.9885	0.0462	0.0192
Bulgur 36.25 yb%	Lewis	K 7.12 × 10^−3^	0.9392	0.0661	0.0306	K 1.12 × 10^−2^	0.9978	0.0132	0.0021
	Henderson and Pabis	K7.92 × 10^−3^ a 1.08	0.9570	0.0600	0.0216	K 1.13 × 10^−2^ a 1.01	0.9981	0.0128	0.0018
	Logarithmic	K 4.08 × 10^−3^ a 1.61 b−5.64 × 10^−1^	0.9830	0.0414	0.0086	K 1.17 × 10^−2^ a 1.00 b 1.56 × 10^−2^	0.9982	0.0131	0.0017
	Midilli	K 4.48 × 10^−4^ a 9.80 × 10^−1^ b 1.10 × 10^−3^ n 1.79	0.9961	0.0197	0.0035	K 8.24 × 10^−3^ a 1.00 b 2.64 × 10^−4^ n 1.08	0.9994	0.0117	0.0012
Bulgur 43.25 yb%	Lewis	K 6.64 × 10^−3^	0.9604	0.0548	0.0420	K 9.68 × 10^−3^	0.9778	0.0004	0.0198
	Henderson and Pabis	K 7.32 × 10^−3^ a 1.07	0.9719	0. 0478	0. 0298	K 9.85 × 10^−3^ a 1.01	0.9782	0.0420	0.0194
	Logarithmic	K 2.48 × 10^−3^ a 2.32 b −1.28	0. 9794	0.0427	0.0218	K 1.06 × 10^−2^ a 9.84 × 10^−1^ b 3.60 × 10^−2^	0.9786	0.0436	0.0190
	Midilli	K 2.55 × 10^−4^ a 9.79 × 10^−1^ b 7.75 × 10^−4^ n 1.60	0.9992	0.0087	0.0007	K 3.07 × 10^−3^ a 9.84 × 10^−1^ b 8.04 × 10^−4^ n 1.31	0.9918	0.0403	0.0146

**Table 5:** The effect of dryer type on the effective diffusion coefficient of bulgur.

		Bulgur 1.75 yb%	Bulgur 36.25 yb%	Bulgur 43.25 yb%
D_eff_ (m^2^/s)	Hot air oven	7.05 × 10^−11^ ± 3.17 × 10^−22 a^	6.86 × 10^−11^ ± 2.05 × 10^−21 a^	6.86 × 10^−11^ ± 4.52 × 10^−21 a^
	Vacuum dryer	7.82 × 10^−11^ ± 7.05 × 10^−22 b^	7.73 × 10^−11^ ± 4.74 × 10^−22 b^	7.73 × 10^−11^ ± 1.81 × 10^−20 b^

Mean values with a row followed by different letters are significantly different (*p* < 0.05).

**Table 6:** The effect of yellow berry percentage on the effective diffusion coefficient of bulgur.

	Samples	Hot Air Oven	Vacuum Dryer
D_eff_ (m^2^/s)	Bulgur 1.75 yb%	7.05 × 10^−11^ ± 3.17 × 10^−22 b^	7.82 × 10^−11^ ± 7.05 × 10^−22 b^
	Bulgur 36.25 yb%	6.86 × 10^−11^ ± 2.05 × 10^−21 a^	7.73 × 10^−11^ ± 4.74 × 10^−22 a^
	Bulgur 43.25 yb%	6.86 × 10^−11^ ± 4.52 × 10^−21 a^	7.73 × 10^−11^ ± 1.81 × 10^−20 a^

Mean values with a row followed by different letters are significantly different (*p* < 0.05).

## Data Availability

The datasets generated for this study are available on request to the corresponding author.
